# Radiofrequency ablation versus laparoscopic hepatectomy for treatment of hepatocellular carcinoma: a systematic review and meta-analysis

**DOI:** 10.1186/s12957-020-01966-w

**Published:** 2020-08-12

**Authors:** Shan Jin, Shisheng Tan, Wen Peng, Ying Jiang, Chunshan Luo

**Affiliations:** 1 Department of oncology, People’s Hospital of Guizhou province, Guiyang City, China; 2 Department of orthopedic, Guizhou Orthopedic Hospital, No. 184, Zhongshan East Road, Nanming District, Guiyang City, 550000 Guizhou Province China

**Keywords:** laparoscopic hepatectomy, radiofrequency ablation, hepatocellular carcinoma, meta-analysis, review

## Abstract

**Background:**

Several randomized controlled trials (RCTs) compared the effects of laparoscopic hepatectomy (LH) and radiofrequency ablation (RFA) for the treatment of hepatocellular carcinoma (HCC), but the results have remained inconsistent. Hence, a meta-analysis and a systematic review of these treatment modalities are necessary to evaluate their efficacy and safety for HCC treatment.

**Methods:**

From the inception of this meta-analysis and review until August 31, 2019, we searched Medline, PubMed, EMBASE, Cochrane Library, China National Knowledge Infrastructure, Wanfang Database, and China Biomedical Literature Database for RCTs involving LH and RFA treatments of patients with HCC. The studies were screened and the data from these articles were extracted independently by two authors. Summary odd ratios (OR) or mean differences (MD) with 95% confidence intervals (CI) were calculated for each outcome with a fixed- or random-effect model. The outcomes for effectiveness evaluations included duration of surgery, estimated bleeding volume, incidence of blood transfusion during surgery, duration of hospital stay, and the outcome for safety included the incidence of cancer recurrence.

**Results:**

Seven RCTs with a total of 615 patients were identified, 312 and 303 of which underwent RFA and LH treatments, respectively. The duration of surgery (MD = −99.04; 95% CI: −131.26–−66.82), estimated bleeding volume (MD = −241.97; 95% CI: −386.93–−97.02), incidence of blood transfusion during surgery (OR = 0.08; 95% CI: 0.02–0.37), and duration of hospital stay (MD = −3.4; 95% CI: −5.22–−1.57) in RFA treatment were significantly lower than those of LH treatment. However, the incidence of cancer recurrence was significantly higher for RFA treatment compared with LH treatment (OR = 2.68; 95% CI: 1.72–4.18).

**Conclusions:**

LH treatment is preferred over RFA treatment with a better radical effect, but RFA treatment is more beneficial with smaller trauma, development of less complications, and shorter operating time when compared with HCC treatment.

## Introduction

Hepatocellular carcinoma (HCC) is a common malignant tumor. It has been reported to cause 200,000 global deaths annually, with approximately half of the deaths occurring in China [1, 2]. With the continued development of early screening and treatment technologies, HCC has evolved from an incurable type of cancer into a treatable, controllable disease over the past decade [3]. Currently, partial hepatectomy and liver transplantation remain as the main strategies of early HCC radical cure. However, liver transplantation cannot be widely conducted due to the lack of allogeneic donors and the inconsistency of transplantation standards [4]. Therefore, liver resection is still the first line of treatment. In general, hepatectomy is divided into two methods, namely, traditional open hepatectomy and laparoscopic hepatectomy (LH). LH provides more advantages than open hepatectomy in terms of less intraoperative blood transfusion and shorter hospital stays with similar short- and long-term survival rates [5–7].

Although surgery provides the best option for patients with hepatic tumors, less than 30% of patients with HCC have an opportunity to undergo surgery [8]. Previous studies [9–11] have demonstrated that local ablation, especially radiofrequency ablation (RFA), can also achieve radical cure effects on HCC, and its short- and long-term survival rates are comparable and even similar to surgery strategies. In addition, RFA treatment has the advantages of mild trauma, low risk of bleeding, and high reproducibility compared with surgery.

In the past decades, minimally invasive technology, with the advancement in technology and the demand for high quality of life, has become increasingly attractive among patients and health care providers, especially in the treatment of small solid tumors [12]. A number of studies have compared the efficacy of open hepatectomy and LH treatment on HCC, but only few of them have focused on the efficacy and safety of RFA and LH treatments for HCC. At present, no guidelines for RFA and LH treatments of liver cancer exist, and a systematic evaluation of the safety and efficacy of RFA and LH treatments for liver cancer is lacking. Therefore, we conducted this meta-analysis and systematic review to evaluate the efficacy and safety of RFA and LH treatments for HCC to provide insights into the clinical treatment of HCC.

## Methods

This meta-analysis was conducted and reported in compliance with the criteria of Preferred Reporting Items for Systematic Reviews and Meta-analysis (PRISMA) [13].

### Search strategy

We attempted to identify the RCTs comparing the effectiveness and safety of RFA and LH. The systematic searches of related literature were performed by two independent reviewers. The databases searched included Medline, PubMed, EMBASE, Cochrane Library, China National Knowledge Infrastructure, Wanfang Database, and China Biomedical Literature Database. The literature search of each database was conducted from the inception date of this study until August 31, 2019. Language restrictions on studies published in English and Chinese were imposed. Randomized controlled trials (RCTs) on the efficacy and safety of RFA and LH treatments for HCC were identified. The following terms and their combinations were searched in related databases: radiofrequency ablation, RFA, laparoscopic hepatectomy, hepatocellular carcinomas, liver neoplasm, laparoscopic, randomized controlled trial, and RCT. The reference lists of previously published reviews were also reviewed and manually searched. Any disagreements were discussed with a third reviewer to reach a consensus.

### Inclusion and exclusion criteria

The inclusion criteria were as follows: (1) HCC is defined as a primary malignancy of the liver that occurs predominantly in patients with underlying chronic liver disease and cirrhosis, and histological examination were performed to diagnose HCC; a single tumor with a diameter of ≤6.5cm has no extrahepatic metastasis; and the liver function should be child grade A or B with no vital organ dysfunction [14]; (2) patients were treated with RFA or LH; (3) study design was RCT; and (4) related surgery detail and outcome indicators were reported. The outcomes for effectiveness evaluations included duration of surgery, estimated bleeding volume, incidence of blood transfusion during surgery, duration of hospital stay, and the outcome for safety included the incidence of cancer recurrence. We attempted to contact the original author by email to obtain the relevant missing data as necessary.

The studies were excluded if the interventions of control and treatment groups remained unclear; no RCT design; the outcomes of interest were not clearly reported; and considerable overlaps between the authors and research centers among the published literature.

### Data extraction

We used a standardized data collection form to extract key information. Any discrepancy in the extraction process was resolved through a consensus. We also attempted to contact authors to obtain additional data or to clarify data of missing details. Two reviewers independently extracted the following information: first author, year of publication, study location, patient population, details of RFA and LH treatments, main outcomes, and study results. The following main outcome measurements were also extracted and analyzed in this meta-analysis: duration of surgery, blood loss, blood transfusion during surgery, length of hospital stay, and incidence of cancer recurrence.

### Quality assessment

The Cochrane collaboration’s risk of bias tool [15] was used by the two independent reviewers to evaluate the methodological quality and risk of bias of the included RCTs, in which any disagreements were resolved by discussion and consensus. This tool was also utilized to examine and measure seven specific domains: sequence generation, allocation concealment, blinding of participants and personnel, blinding of outcome assessment, incomplete outcome data, selective outcome reporting, and other issues. Each domain was classified as low risk of bias, high risk of bias, or unclear risk of bias according to the judgement criteria.

### Data analysis

All statistical analyses were performed using RevMan 5.3 software. Data were encoded and double-checked by the two reviewers. Data syntheses and interpretations were also performed by the two authors to ensure the accuracy of results. Binary outcomes were presented as Mantel–Haenszel-style odds ratios with 95% confidence interval (CI). Continuous outcomes were reported as mean differences (MDs). A fixed-effect model was adopted in cases of homogeneity (P-value of χ^2^ test > 0.10 and *I*^2^ < 50%), whereas a random-effect model was used in cases of apparent heterogeneity (P-value of χ^2^ test > 0.10 and *I*^2^ ≥ 50%) [16]. Publication bias was evaluated using funnel plots, and asymmetry was assessed via Egger regression test. For funnel plot asymmetry, P < 0.1 was considered significant.

## Results

**Fig. 1:**
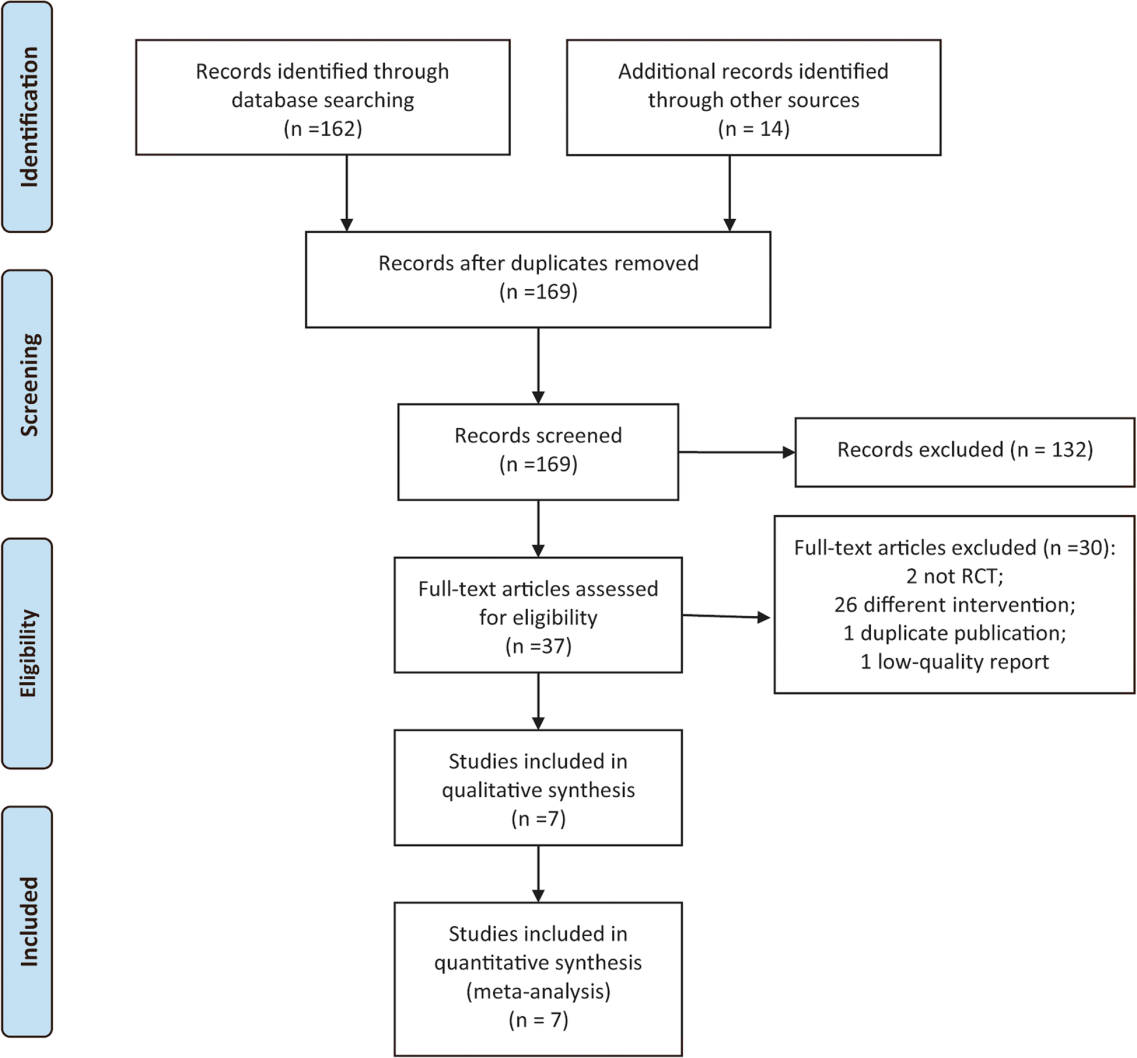
Flow chart of study selection

The initial literature search yielded 176 studies. The number of records, after duplicated articles were removed, was 169. Furthermore, a total of 132 studies were excluded after screening the titles and abstracts. A total of 37 studies were reviewed for eligibility by scrutinizing full-text articles. Eventually, seven RCTs [10, 17–22] met the inclusion criteria and were included for meta-analysis. The process of study selection is presented in Fig. 1.

### Characteristics of included RCTs

The characteristics of the seven included RCTs are presented in Table 1. These seven RCTs enrolled a total of 615 randomized participants, with 312 and 303 patients received RFA and LH treatments, respectively. The sample sizes varied from 46 to 156 patients. Five studies [10, 18–21] were conducted in China. The mean age of patients varied from 48 to 73 years old. The results from most included studies supported the use of LH treatment in patients with uterine fibroids.

**Table 1:** The characteristics of included RCTs

Studies	Group	Participants	Age(years)	Gender (male/female)	Child grade A/B	Conclusions
Casaccia 2017 [17]	RFA	22	60.82±7.25	16/6	20/2	The results confirm the superiority of Hepatic resection on thermoablation in the treatment of Small HCC in selected patients
	LH	24	63.58±9.55	18/6	22/2	
He 2016 [18]	RFA	38	54.1±11.2	30/8	28/10	Radiofrequency ablation has more favorable clinical efficacy, less pain and better quality of life of patients than laparoscopic hepatectomy
	LH	41	52.7±9.4	28/13	30/11	
Lai 2016 [10]	RFA	33	62.8±11.3	23/26	29/4	LH may provide better curative effects than pRFA without increasing complication rates.
	LH	28	56.5±12.6	22/27	24/4	
Song 2015	RFA	78	48 (43, 58)	70/8	76/2	There was no difference between LH and RFA in terms of OS in patients with a single, small HCC.
	LH	78	48 (44,57)	70/8	78/0	
Wang 2017 [20]	RFA	65	66.47±16.13	36/29	47/18	Compared with LH, pRFA has higher success rate in the treatment of SCC.
	LH	61	65.43±15.56	35/26	46/15	
Xu 2017 [21]	RFA	35	57.06±12.6	27/8	32/3	RFA and LH have similar efficacy for small primary liver cancer
	LH	30	52.83±9.4	25/5	29/1	
Yazici 2016	RFA	41	73.7±6.7	24/17	N/A	LH was tolerated as well as RFA in elderly patients with similar comorbidities
	LH	41	72.6±5.0	25/16	N/A	

Notes: HCC, hepatocellular carcinoma; LH, laparoscopic hepatectomy; pRFA, percutaneous radiofrequency ablation; OS, overall survival; N/A, not available

### Quality evaluation

**Fig. 2:**
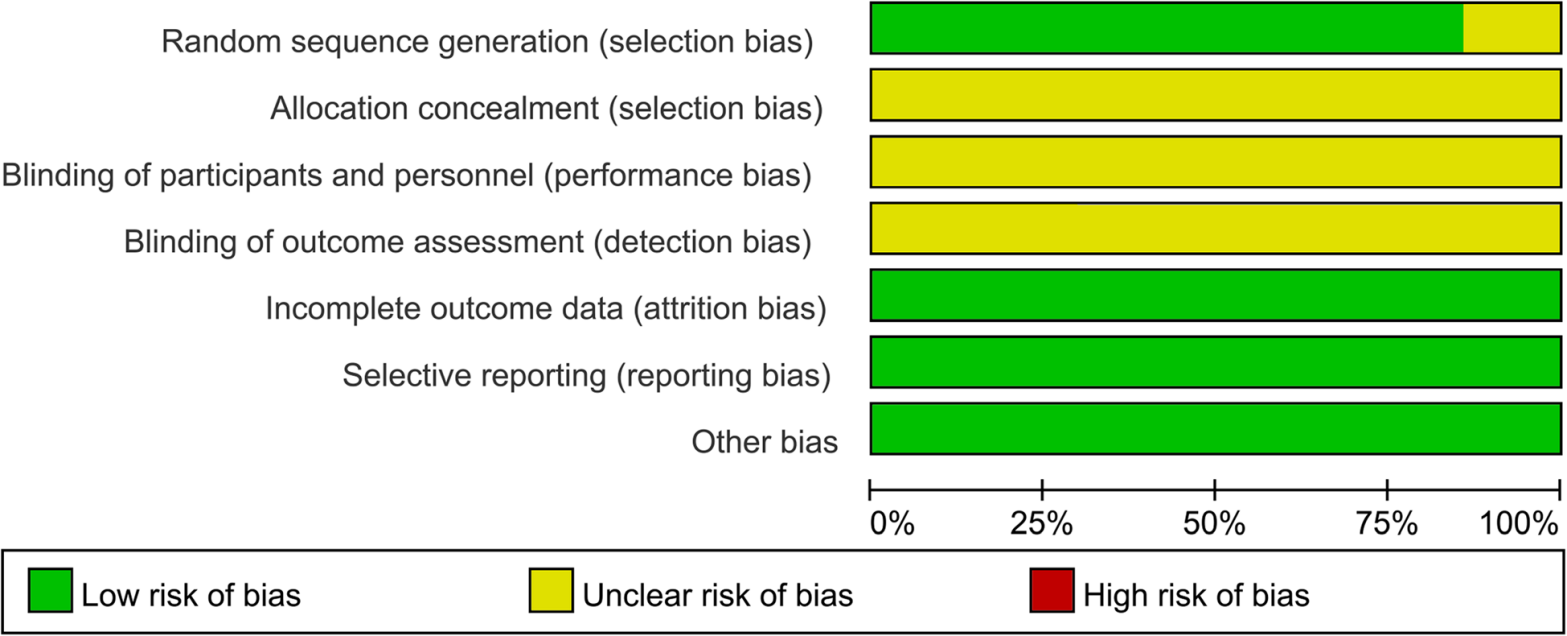
Risk of bias graph

**Fig. 3:**
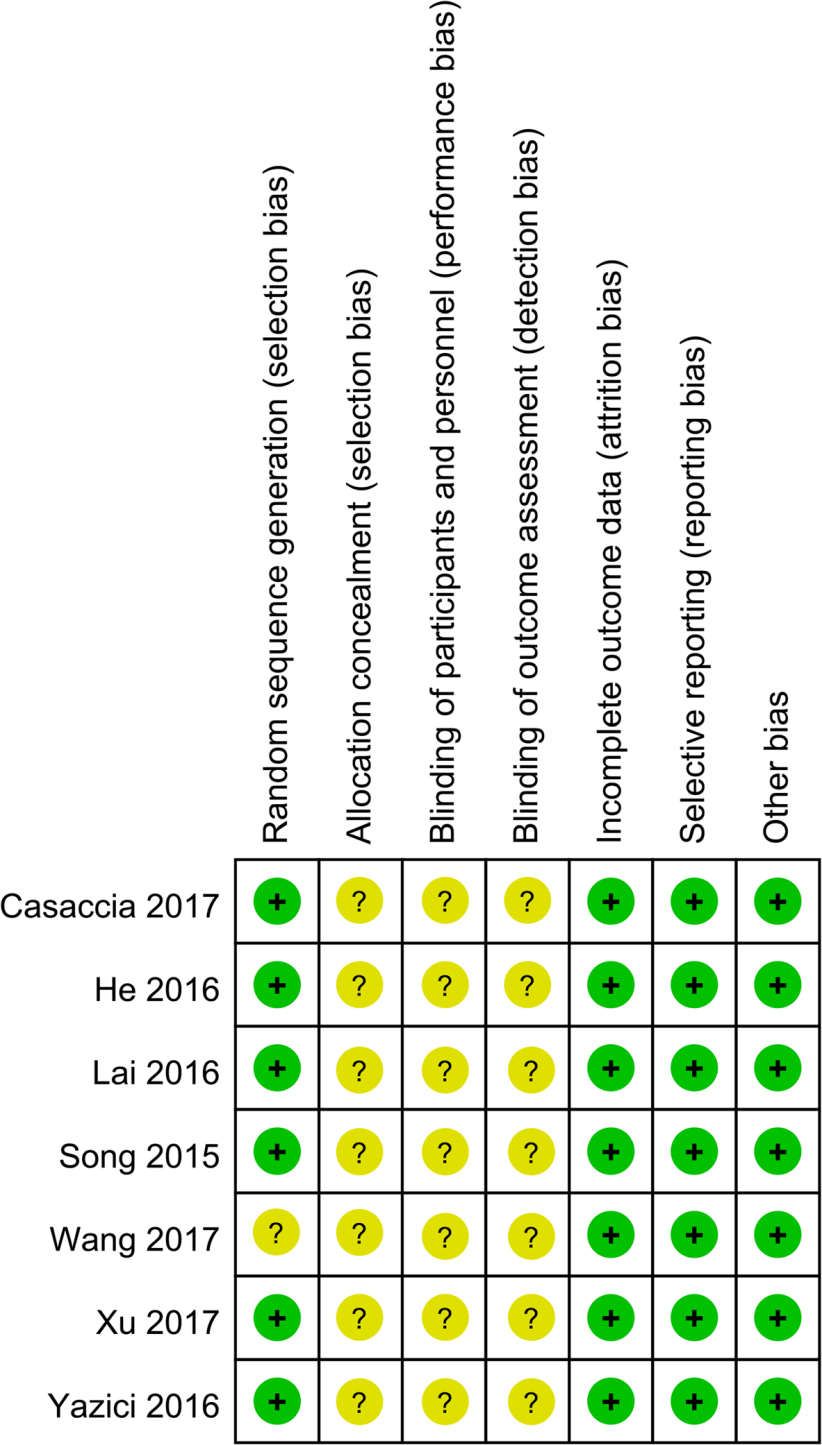
Risk of bias summary

The results of the methodological quality evaluation are presented in Figs. 2 and 3. Following strict judgments of each included RCT according to the Cochrane handbook, we found that no RCT provided a detailed description of the methods used to produce a random sequence, although all included RCTs mentioned randomization. Moreover, all included RCTs did not report allocation blinding or personnel blinding. All included studies did not report related information for the blinding of outcome assessment. No selective reporting or other remarkable biases among the seven included RCTs were observed.

### Outcomes

#### Duration of surgery

**Fig. 4:**
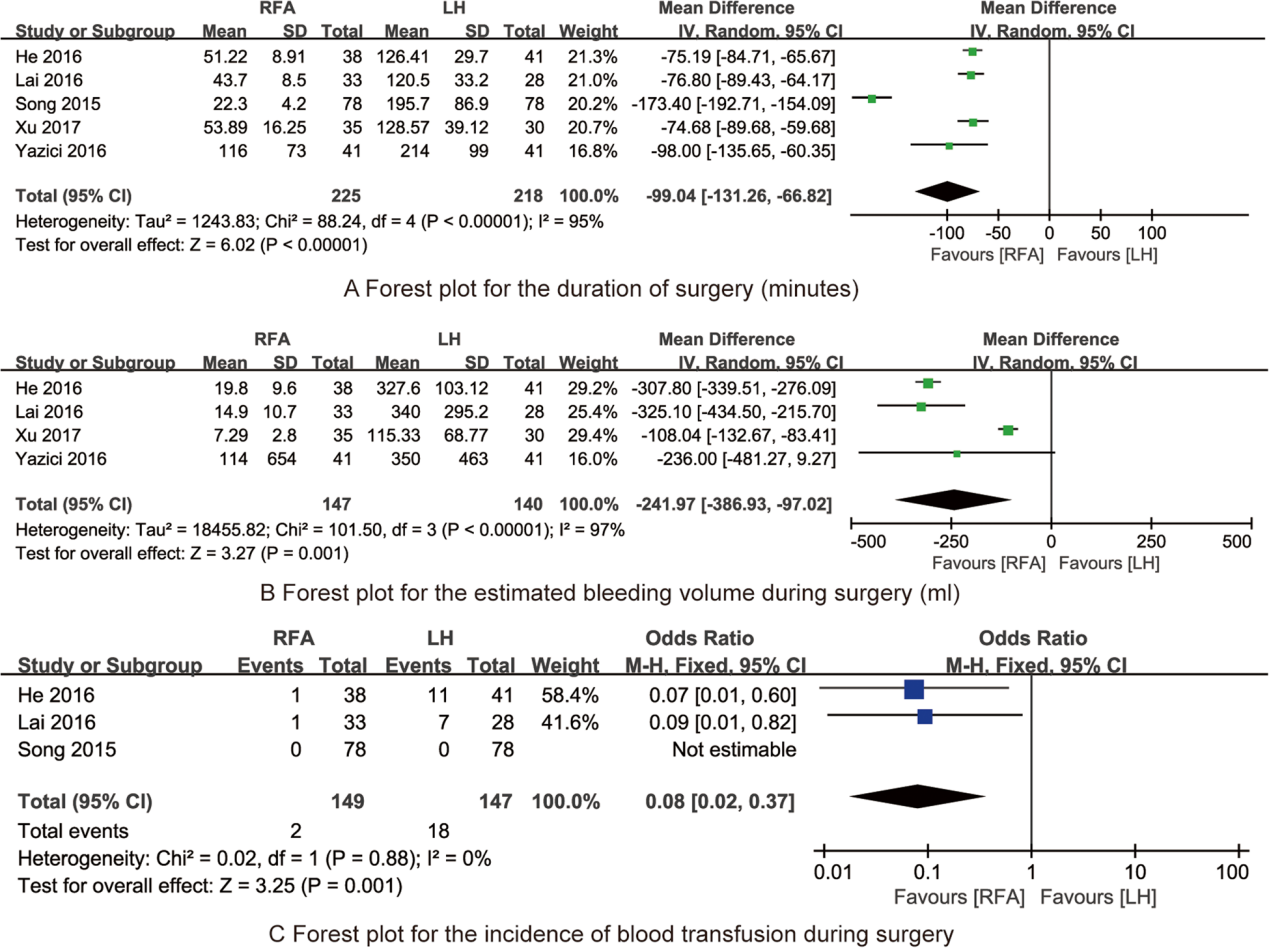
Forest plot for synthesized outcomes

Five studies [10, 18, 19, 21, 22] reported the duration of surgery among the RFA and LH treatments. The pooled data from the five RCTs revealed that the duration of surgery for RFA treatment was significantly shorter than that of LH treatment (MD = −99.04; 95% CI: −131.26–−66.82; *P* < 0.001; *I*^*2*^ = 95%; Fig. 4a).

#### Estimated bleeding volume during surgery

Four studies [10, 18, 21, 22] stated the estimated bleeding volume during surgery among the RFA and LH treatments. The pooled data from the four RCTs showed that the estimated bleeding volume during surgery for RFA treatment was significantly lesser than that of LH treatment (MD = −241.97; 95% CI: −386.93–−97.02; *P* < 0.001; *I*^*2*^ = 97%; Fig. 4b).

#### Incidence of blood transfusion during surgery

Three studies [10, 18, 19] reported the incidence of blood transfusion during surgery among the RFA and LH treatments. The pooled data from the three RCTs indicated that the incidence of blood transfusion during surgery for RFA treatment was significantly lower than that of LH treatment (OR = 0.08; 95% CI: 0.02–0.37; *P*=0.001; *I*^*2*^ = 0%; Fig. 4c).

#### Duration of hospital stay

**Fig. 5:**
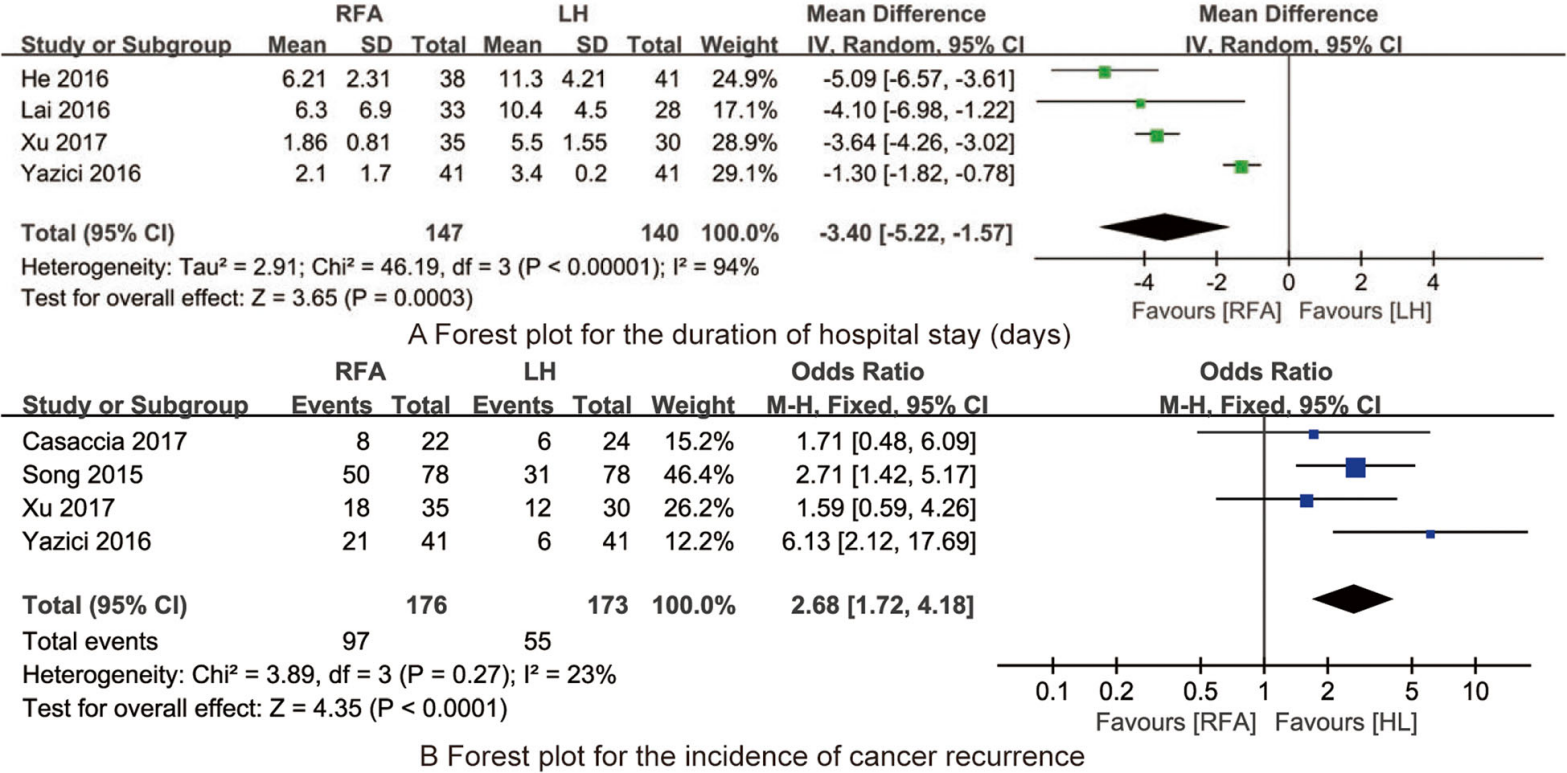
Forest plot for synthesized outcomes

Four studies [10, 18, 21, 22] reported the duration of hospital stay among the RFA and LH treatments. The pooled data from the four RCTs suggested that the duration of hospital stay for RFA treatment was significantly shorter than that for LH treatment (MD = −3.4; 95% CI: −5.22–−1.57; *P*<0.001; *I*^*2*^ = 94%; Fig. 5a).

#### Incidence of cancer recurrence

Four studies [17, 19, 21, 22] reported the incidence of cancer recurrence among the RFA and LH treatments. The pooled data from the four RCTs indicated that the incidence of cancer recurrence for RFA treatment was significantly higher than that for LH treatment (OR = 2.68; 95% CI: 1.72–4.18; *P*<0.001; *I*^*2*^ = 23%; Fig. 5b).

### Subgroup and sensitivity analyses

Subgroup analyses were not performed in this meta-analysis because the details of RFA and LH treatments of the included studies were considerably different. We aimed to evaluate publication bias by using a funnel plot if 10 or more RCTs were included in an outcome meta-analysis. However, due to the limited number of included RCTs, we could not perform funnel plot.

Sensitivity analyses, which investigate the influence of one study on the overall risk estimate by removing one study in each turn, suggested that the overall risk estimates were not substantially changed by any single study.

## Discussion

Related guidelines state that liver transplantation, hepatectomy, and RFA treatment can all achieve clinical cure for liver cancer [23, 24]. In China, liver transplantation cannot be widely implemented due to the large number of patients with HCC and limited donors [25, 26]. Therefore, LH and RFA treatments are still the preferred treatments for patients with liver cancer. However, no consensus has been achieved on the choice between these two minimally invasive treatments. The present meta-analysis focused on the therapeutic effects and safety of RFA and LH treatments. Results showed that RFA and LH treatments can effectively cure HCC with a diameter of ≤6.5 cm, but RFA treatment has a shorter operation time, lesser intraoperative blood loss, shorter hospital stay, and lesser risk of blood transfusion than LH treatment. Nevertheless, patients with HCC who received RFA treatment have higher incidence of recurrence compared with those who underwent LH treatment.

Percutaneous or laparoscopic approaches for RFA treatment are generally chosen to ablate and destroy the lesion and surrounding tissue under the guidance of color Doppler ultrasound and CT [27]. Performing hemostasis or suture on the liver as hepatectomy needs is not necessary. The procedure is relatively simple and easy, thus substantially reducing the operating time [28]. For tumors in the liver segment where surgical resection is difficult, the target part can be relatively easily reached by the radiofrequency needle, and the tissue around the puncture site is less damaged [29]. Damaging the adjacent tissues, such as blood vessels and bile ducts, is relatively difficult even if the puncture is repeated [30]. Compared with LH treatment, bleeding is considerably reduced, and the possibility of transfusion is low during and after surgery in RFA treatment [31]. In contrast to RFA treatment, LH treatment must deal with the liver section that demands greater skills and dexterity from surgeons and causes a higher risk of related complications, such as postoperative hemorrhage, bile leakage, ascites, pleural effusion, and lung infection [32]. Patients treated with RFA may also experience postoperative bleeding, pain, and pleural effusion, but the incidence of complications is reportedly lower than that of LH treatment [33, 34]. Hence, the average hospital stay for patients treated with RFA is significantly shorter than that for patients treated with LH.

However, RFA treatment may have three-dimensional leak in large or irregularly shaped tumors, resulting in residual lesions [35]. Moreover, the extent of thermal ablation is generally limited, although this can be compensated for by repeated operations, but the possibility of omission exists [36]. Furthermore, after the arterial blood supply of tumor is destroyed by thermal coagulation, subsequent follow-up treatment, such as transcatheter arterial chemoembolization, is highly required [37, 38]. We believe that these observations are the reasons tumors are more likely to recur after RFA treatment. Previous studies [39–41] compared the efficacy of RFA treatment and radical hepatectomy (including open surgery, total laparoscopic surgery, and laparoscopic assisted surgery) for HCC. Results showed that the tumor-free survival time of RFA-treated patients is considerably shorter than that of the operation group, but no significant difference in in-hospital mortality and overall survival between the two groups was observed. The perioperative complications of the patients in the RFA treatment group are lesser than those in the surgery group, and the length of hospital stay in the RFA treatment group is remarkably shorter than that in the surgery group [42, 43]. Therefore, hepatectomy may be a better control for the recurrence of HCC and to ensure a long tumor-free survival.

Several limitations must be considered in the present meta-analysis. First, the trials included in this meta-analysis were influenced by physicians and patients because of the choice of surgical methods. Therefore, the grouping method might not be completely randomized or double-blind controlled. Although all trials reported the case allocation and factors affecting prognosis, achieving a complete matching of baseline data between two groups is difficult. Second, several included studies had generally small sample sizes, and thus these works may have suffered from certain publication bias. Third, several studies reported that RFA treatment is comparable with hepatectomy for the treatment of HCC with a diameter of less than 3 cm. However, this assertion is controversial when the diameter of HCC is more than 3 cm but less than 5 cm. RFA treatment is less effective when HCC is more than 5 cm. We included patients with a tumor diameter of ≤6.5 cm; however, due to the limited information, we could not perform further sub-group analyses. Further investigations on the treatment of HCC with different tumor diameters are needed in the future.

In conclusion, LH and RFA treatments are radical minimally invasive treatments for early-stage liver cancer. RFA treatment results in shorter operation time, lesser intraoperative blood loss, shorter hospital stay, and lesser risk for blood transfusion than LH treatment. Nevertheless, patients with HCC who received RFA treatment are more likely to experience cancer recurrence compared with those who underwent LH treatment. LH treatment see to have better curative effect. But RFA treatment has the advantages of less trauma, fewer complications, and shorter surgery time. Hence, treatment selection should be based on the general condition of the patient.

## Supplementary information

**Additional file 1.** PRISMA 2009 Flow Diagram

## Data Availability

All the data are available and shared in the manuscript.
