# Supplementary material for: Radiofrequency ablation versus laparoscopic hepatectomy for treatment of hepatocellular carcinoma: a systematic review and meta-analysis
Source: World J Surg Oncol. 2020 Aug 12;18:199. doi: 10.1186/s12957-020-01966-w (PMC7425008; doi:10.1186/s12957-020-01966-w)
Supplement: Supplementary file 1 — Additional file 1. PRISMA 2009 Flow Diagram [file 12957_2020_1966_MOESM1_ESM.doc]

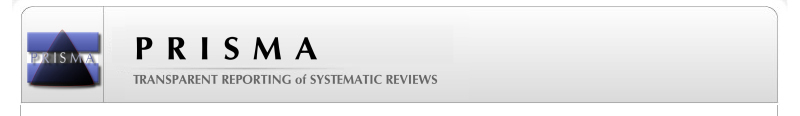
**PRISMA 2009 Flow Diagram**

**Screening**

**Included**

**Eligibility**

**Identification**

Records identified through database searching
(n =162)

Additional records identified through other sources
(n = 14)

Records after duplicates removed
(n =169)

Records screened
(n =169)

Records excluded (n = 132)

Full-text articles assessed for eligibility
(n =37)

Full-text articles excluded (n =30):

2 not RCT;

26 different intervention;

1 duplicate publication;

1 low-quality report

Studies included in qualitative synthesis
(n =7)

Studies included in quantitative synthesis (meta-analysis)
(n = 7)
